# Warming but not Food Limitation Alters Metabolism During Larval Development in Crown‐of‐Thorns Sea Stars (*Acanthaster* cf. *Solaris*)

**DOI:** 10.1002/ece3.73934

**Published:** 2026-07-08

**Authors:** Ronan T. Hill, Amanda Pettersen, Maria Byrne

**Affiliations:** ^1^ University of Sydney Camperdown New South Wales Australia

**Keywords:** climate change, COTS, echinoderm, larvae, metabolic suppression, respiration

## Abstract

Marine ectotherms are vulnerable to physiological challenges wrought by climate change‐driven ocean warming (OW). It is important to understand the impacts of OW on fundamental processes in keystone species such as the corallivorous crown‐of‐thorns sea star (CoTS; *Acanthaster* cf. *solaris*), which poses a key threat to coral reefs. The planktonic life phase of CoTS may benefit from OW through faster development, but increasing temperature can be metabolically costly. We tested whether CoTS larvae can maintain a positive energy budget under warming to offset increased energetic demands within their thermal tolerance range. We reared fed and unfed CoTS larvae at four temperature treatments (control: 26°C; warm: 28°C, 30°C, 32°C) based on current and projected OW and measured metabolic rate (oxygen consumption, $\dot{V}O_{2}$) across development to the late larval stage. As predicted, warming increased metabolic rates, with oxygen consumption higher at 30°C and 32°C, than at the control 26°C. High mortality occurred at 32°C in late bipinnaria (14 days), likely due to cumulative heat stress over time. In the 28°C and 30°C treatments oxygen consumption of bipinnaria (14–21 days) and brachiolaria larvae decreased, potentially due to metabolic suppression. The resilience of unfed larvae (to 21 days) and the similar metabolic rates of fed and unfed larvae to Day 14 indicate that the energetic requirements for larval maintenance and growth were provided by maternal (egg) nutrients, a resilience trait of CoTS. Our results indicate that under increased temperature, CoTS larvae can maintain positive energy budgets. While the presence of food is not critical for development to late bipinnaria, reduced growth in warm treatments in the absence of food may have fitness consequences. Our results highlight the need to understand the energy budget across the entire life cycle of CoTS.

## Graphical abstract

This work investigates how two factors (food and temperature) affect the metabolic rates of the larval stages of ecologically important coral‐eating crown‐of‐thorns sea star (Acanthaster sp.). Warming increased metabolic rates, with oxygen consumption at raised temperatures compared to control. After accounting for larval size, feeding had no effect on metabolic rates.

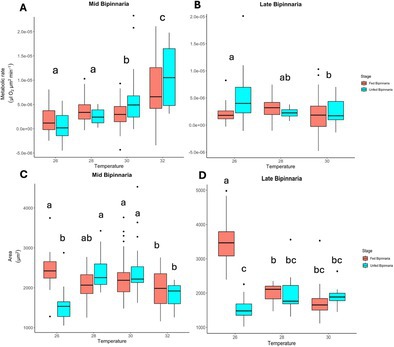

## 1 Introduction

Global warming is imposing unprecedented constraints on animal physiology, threatening the capacity of metabolic function to support fundamental biological processes (Rohr et al. 2018; *IPCC* 2023). Environmental warming affects all aspects of ectotherm biology, including metabolism, development, growth, and overall fitness (Rohr et al. 2018). Due to their complex life histories, the physiology of ectothermic marine invertebrates may be especially vulnerable to ocean warming (OW) and marine heatwaves (Somero 2012; McGeady et al. 2021). The distribution of marine species with planktonic larvae is strongly influenced by larval thermal tolerance, particularly as early life stages are considered to be the most sensitive to thermal stress (Sunday et al. 2011; Przeslawski et al. 2015; Byrne et al. 2022). In a warming world, understanding the thermal physiology of early life stages is important for predicting the persistence of marine species in their native range, as well as their capacity to range extend (Byrne et al. 2022; Pottier et al. 2026).

Metabolic rates reflect the ‘pace of life’ and, as key physiological traits, they can provide important insights into how species may cope with environmental change (Carey et al. 2016; Pettersen et al. 2018; Vorsatz et al. 2021; Byrne et al. 2023). Warming ocean temperatures increase metabolic rates and thereby energy demands, reducing nutritive reserves and potentially the capacity to maintain positive energy budgets (Vorsatz et al. 2021; Moreira et al. 2022; Hardison and Eliason 2024). To some extent, increased feeding stimulated by warming may offset this increase in energetic demand, as shown in studies of oysters (Haure et al. 2003), copepods (Tyrell et al. 2020), mussels (Lee et al. 2021) and sea urchins (McEdward 1985; Brockington and Clarke 2001; Carey et al. 2016; Harianto et al. 2018; Munstermann et al. 2026).

Marine ectotherms are known to suppress metabolic activity as an adaptive response to temperatures outside their thermal niche (Guppy and Withers 1999). In stressful environments, where temperature increase outpaces the supply of food, ectotherm populations may face ‘metabolic meltdown’ (Huey and Kingsolver 2019). Metabolic suppression, involving a decrease in oxygen consumption, may be a strategy to offset the effects of temperature on metabolism. While metabolic suppression can be an advantage to coping with acute thermal stress, prolonged metabolic suppression can reduce growth, reproduction and survivorship (Guppy and Withers 1999; Pörtner and Knust 2007; Peck et al. 2008, 2009; Harianto et al. 2018).

Understanding the impacts of environmental warming on physiology and thermal tolerance is especially important for ecologically influential species, such as the crown‐of‐thorns sea star (CoTS: *Acanthaster* cf. *solaris*), a corallivorous species that poses a major threat to coral reefs (Pratchett et al. 2014; Deaker and Byrne 2022a; Foo et al. 2024). Under combined stressors of warming and low food, larval and early juvenile CoTS exhibit high resilience (Kamya et al. 2014, 2016; Lamare et al. 2014; Deaker and Byrne 2022b; Byrne et al. 2023; Mos et al. 2024). The larvae show high survival and normal development (3 days post fertilisation—dpf) across a broad thermal range (24°C–32°C), including temperatures representative of heatwave conditions (Lamare et al. 2014; Kamya et al. 2014). By 10 dpf deleterious effects were evident in developmental arrest at 30°C and 100% mortality at 32°C (Kamya et al. 2014). Although it has not been examined in CoTS, the presence of increased food availability may enhance larval survival in warm conditions as seen for sea urchins (Munstermann et al. 2026). It has been suggested that CoTS larvae may be resilient to OW and so will remain a threat to coral reefs (Uthicke et al. 2015).

As a species adapted to low nutrient oligotrophic tropical seas, CoTS larvae are resilient to food scarcity (Olson 1987; Caballes et al. 2016; Mos et al. 2024). This resilience is suggested to be facilitated by the extensive energetic reserves in their unusually large eggs (~250 μm diameter) (Caballes et al. 2016). These eggs are larger than those of other echinoderms with planktotrophic larvae and likely provide a nutritive buffer for larvae and allow for a long facultative feeding period (FFP) when they can develop in the absence of exogenous food (Byrne et al. 2008; Deaker et al. 2019). The larvae can also avail of pulses of phytoplankton to increase their size and condition, reducing time to settlement (Wolfe et al. 2015a). This nutritive flexibility is suggested to be a resilience trait that contributes to the success of CoTS and generation of population outbreaks of this sea star (Deaker and Byrne 2022a). The impact of the presence of exogenous food on larval metabolism across a range of rearing temperatures has not been investigated for CoTS, although high food conditions are suggested to promote outbreaks (Uthicke et al. 2015).

We conducted the first study of metabolic rates through the planktonic life stages of the *Acanthaster* cf. *solaris*. The larvae were reared in a range of temperatures (26°C–32°C), with the 28–30 reflecting the level of warming (1.5°C, 2°C–4°C) predicted under ocean warming (*IPCC* 2023), with the 32°C treatment known to cause larval abnormality and mortality (Lamare et al. 2014). We measured oxygen consumption rates as a proxy for metabolism on individual larvae using closed micro‐respirometry. This method has been used in studies in a range of marine larvae (Pettersen et al. 2015; Schwemmer et al. 2020; Vorsatz et al. 2021) and juvenile CoTS (Deaker and Byrne 2022b). We hypothesised that metabolic rates of CoTS larvae would increase with deleterious impacts seen at 32°C—the suggested thermal tolerance limit of CoTS larvae (Lamare et al. 2014). Access to exogenous food promotes larval growth in CoTS (Wolfe et al. 2015a) with increased ingestion rates at higher algal densities (Patel et al. 2024). We thus hypothesised that the presence of exogenous food would promote higher metabolic rates and growth relative to those reared in the absence of food, as observed in other echinoderm larvae (Pace and Manahan 2007; Peck et al. 2008; Ginsburg and Manahan 2009; Rendleman et al. 2018). We also expected that growth and respiration of CoTS larvae would increase in the warm treatments. Our study provides insight into how OW may affect the planktonic life stage of CoTS and thereby influence their potential to generate outbreaks.

## 2 Materials and Methods

### 2.1 Larval Rearing

Adult CoTS collected near Cairns (northern Great Barrier Reef, GBR) in November 2023 were shipped to the Sydney Institute of Marine Science where they were maintained in flow‐through aquaria supplied with filtered seawater (FSW 5.0 μm) at 26°C–27°C, the temperature near the collection site. Although the taxonomy of the west Pacific species of CoTS that occurs on the GBR remains to be determined due to lack of type specimen and a reliable type locality (Haszprunar and Spies 2014; Hutchings et al. 2025), we apply the name in current use *Acanthaste*r cf. *solaris*. Small pieces of ovary and testis were dissected from 2 male and 2 female sea stars. The ovaries were rinsed in 1 μm FSW and placed in 1 μM 1‐methyl adenine in FSW to obtain fertile eggs. The sperm of the two males were combined and used to fertilise the eggs using routine methods (Clements et al. 2022). The embryos were reared for ~12 h at 25°C–26°C in 5 L beakers before being placed in experimental treatments. Prior to hatching, embryos were placed into 1 L beakers of 1 μm FSW at 26°C in a constant temperature room. For warm treatments, the temperature of culture beakers was increased at 1°C intervals approximately every 3 h to the target temperature levels 28°C, 30°C and 32°C in water baths. For the fed cultures, larvae were provided *Proteomonas sulcata* (20,000 cells mL^−1^) every 2 days following water renewal at experimental temperature using reverse aspiration. For the non‐fed cultures, water was changed in parallel, but algae were not added.

### 2.2 Developmental Stages Measured

**FIGURE 1:**
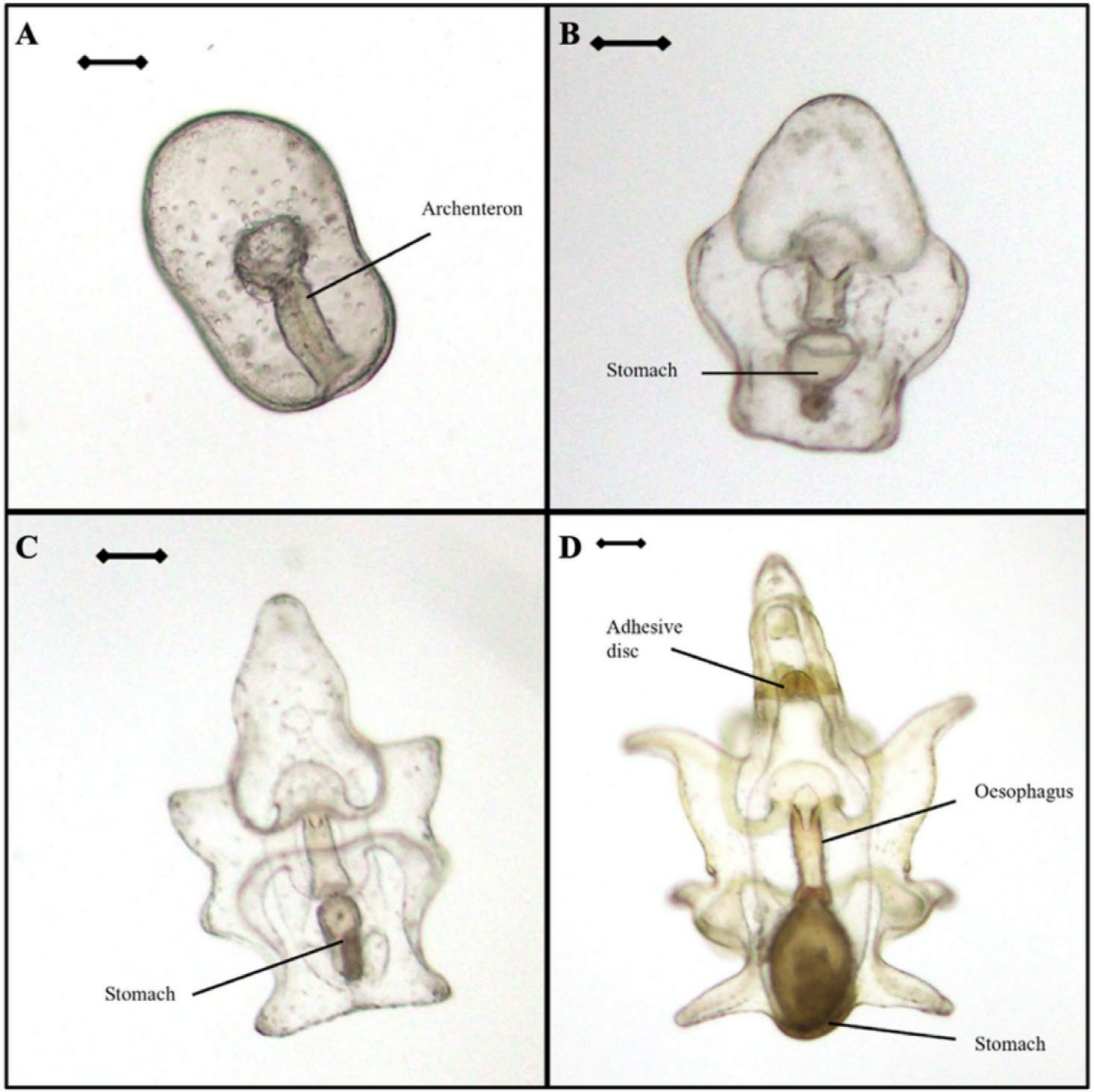
Developmental stages of *Acanthaster* cf. *solaris* used for respirometry. (A) Gastrula, (B) Early bipinnaria, (C) Late bipinnaria, (D) Brachiolaria. The adhesive disc shows the location of the attachment complex used for settlement, Scale = 50 μm.

Metabolic rate of individual gastrulae (~24 h post fertilisation—hpf), early bipinnaria (just prior to feeding, ~3 dpf), feeding stage bipinnaria (7–14 dpf) and brachiolaria (21 dpf) were measured (see Table A1 for the stages investigated). The metabolic rates of fed and unfed early and mid bipinnaria from the at 26°C, 28°C, 30°C and 32°C treatments were measured on Day 7 and 14. By the late bipinnaria (14 days) stage only cultures reared at 26°C, 28°C and 30°C (fed and unfed) could be tested because the 32°C cultures had 100% mortality. In addition, the unfed cultures died before reaching brachiolaria in all temperature treatments. For the brachiolaria stage (21 dpf) only larvae from fed cultures reared at 26°C and 28°C were available for respirometry. Successful development to the brachiolaria was indicated by the formation of the attachment complex which is essential for larval settlement (Figure 1).

Metabolic rates were measured as the rate of oxygen consumption ($\dot{V}O_{2}$) using closed respirometry following Pettersen et al. (2015). Individual larvae were placed into separate 80‐μL wells within 24‐well glass microplates (Loligo Systems ApS, Viborg, Denmark) fitted with a 3 mm oxygen sensor spot (Figure A2). Fed larvae were hand‐picked and placed in clean FSW and starved for 24 h prior to measurement to ensure they were in a post food‐absorptive state to reduce noise in oxygen consumption due to digestion. Examination of the larvae indicated that their stomachs were empty. The 80‐μL wells were filled with pasteurised FSW (microwaved for 8 min 24 h prior), sealed and checked to ensure that no air bubbles were present. The microplates were secured onto SensorDish using grip plastic and weight, then placed into a 1 L water bath with recirculating water connected to a 30 L header tank at the measurement temperature (26°C, 28°C, 30°C, 32°C). Percentage oxygen saturation was measured at 1‐min intervals using PreSens Sensor Dish reader (SDR) software (v4) for approximately 3.5 h or until there was a 20% decrease in oxygen saturation. Empty wells (*n* = 6) without a specimen were used as controls to check for background changes in oxygen levels. The wells did not require stirring or movement of water for the optical sensors to accurately measure oxygen concentration (Irwin and Davenport 2006). Since larvae were mobile during measurements, it is unlikely that localised oxygen depletion occurred in the wells.

**FIGURE A1:**
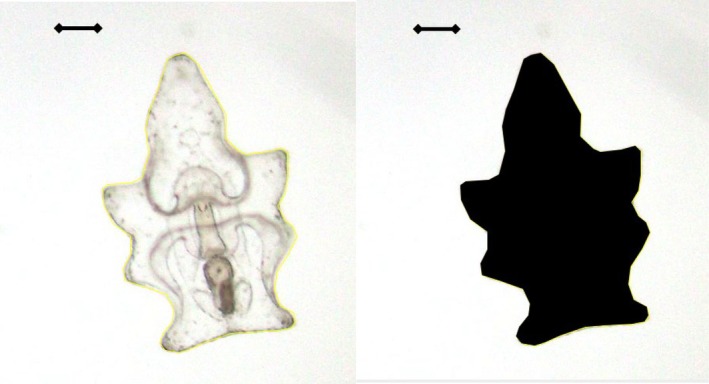
(A) Tracing of late bipinnaria larvae CoTS seen in faint yellow line (B) Total area calculated from trace in μm^2^. Images were processed in ImageJ (ImageJ version 1.54 K browser edition), scale bar is equal to 50 μm.

**FIGURE A2:**
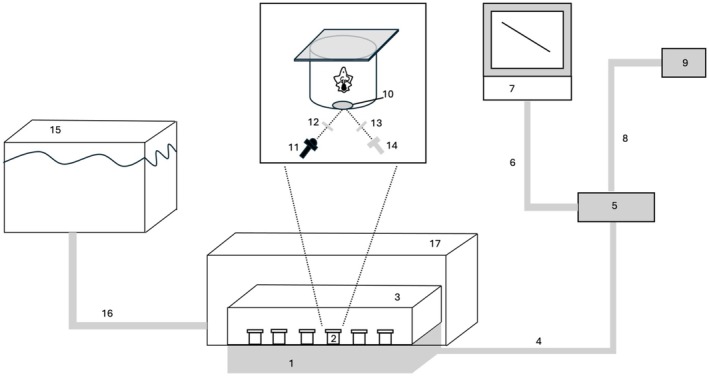
Schematic figure of experimental setup for optical measurement of dissolved oxygen in miniaturised respiration chambers (80 μL) for individual respirometry on *Acanthaster* cf. *solaris* larvae: (1) 24‐channel sensor dish, (2) respiration chamber, (3) dark incubator, (4) RJ 45 cable, (5) splitter, (6) PC connection, (7) computer, (8) power adapter connector, (9) power adapter, (10) planar optode, (11) light‐emitting diode, (12) excitation filter, (13) emission filter, (14) photodiode, (15) water tub at treatment temperature, (16) water connector, (17) flow‐through water bath for microplate. Schematic figure modified from Köster et al. (2008).

To determine the effect of body size on metabolic rates, all embryos and larvae were photographed prior to being placed in the respirometry wells using an Olympus compound microscope with an attached DP23 digital microscope camera and Olympus CellSens software. The surface area of the embryos and larvae was measured by tracing to the nearest μm^2^ using a 1 mm scale bar and ImageJ software (ImageJ version 1.54 K browser edition) (Figure A1).

The $\dot{V}$O_2_ for individual gastrulae and larvae were calculated by extracting the slope for all wells selecting for the most linear sections of each slope utilising *RespR* package in R, with the initial 30 min of measurements removed to allow for accurate depiction of data (Harianto et al. 2019). The average blank slope was calculated based on the control wells and then subtracted from the experimental wells. $\dot{V}O_{2}$ (μLO_2_min^−1^) was calculated per Equation (1),

(1) $$\dot{V}O_{2}=-1\;\left(\mathrm{ma}-\mathrm{mb}\right)/100\times V\times bO_{2}$$

where m_a_ is the rate of change of O_2_ saturation for experimental wells (% per hour), m_b_ is the rate of change of O_2_ saturation for control wells (% per hour), *b*O_2_ is the oxygen capacitance of sea water at the measured temperature (Cameron 1986—see Appendix table 2.1), and V is the water volume in the well.

### 2.3 Statistical Analysis

All data were analysed using R (Ver. 5.1.13). To determine the effect of temperature and feeding regime on metabolism, data for each developmental stage were analysed separately. This was done as not all stages were represented at each temperature and feeding regime (see Table A1). Larval area and metabolic rate data were natural log transformed. Model assumptions were assessed with inspection of diagnostic plots using *dHARMa* (Hartig 2024) and *ggplot2* (Wickham 2016) packages. Linear mixed effects models were fitted using ‘lmer’ function within the *lme4* package (Bates et al. 2015). The *lme4* package in R allows for non‐independent measures on the same individuals, allowing larval area to be linked to the respective individual.

For the gastrula stage, metabolism data did not meet the assumption of homogeneity of variance (HOV), although they were normally distributed. As a result, the data were analysed by a Kruskal‐Wallis rank sum test with temperature as the categorical predictor. Post hoc pairwise comparisons between temperature levels were conducted using Dunn's test with Bonferroni correction to control for multiple comparisons. Size was not included in the model for gastrulae as there were no among‐individual differences in size at this stage. A small subset of individuals was measured for averaging size for data visualisation (*n* = 8).

The data for early bipinnaria and brachiolaria were analysed by linear mixed effects models with temperature, size, and their interaction included as fixed effects and ‘SDR reader ID’ included as a random effect. The models for early bipinnaria and brachiolaria met assumptions of HOV and normality. These data were analysed using type III ANOVA with Satterthwaite's approximation for denominator degrees of freedom. Post hoc analyses were conducted for metabolic rate models utilising *emmeans* (Lenth 2025) adjusted for TukeyHSD.

The data for the middle and late bipinnaria were initially analysed using a linear mixed effects model with three fixed effects (size, temperature and feeding treatment) and one random effect (SDR reader ID). These analyses showed that there was no effect of food (Table A2). As a result, data for fed and unfed treatments were analysed together for each temperature using a linear mixed effects model with two fixed effects (size and temperature) and one random effect (SDR reader ID). Assumptions of HOV and normality were met. Statistical significance of fixed effects was assessed using type II ANOVA. Post hoc analyses were conducted for all metabolic rate models utilising *emmeans* adjusted for TukeyHSD.

Excluding gastrula, candidate models and interactions (Tables A3, A4, A5) were ranked using conditional Akaike Information Criterion (AICc) for all developmental stages using the R package *MuMIn* (Bartoń 2025). All combinations of fixed effects and interactions were considered.

To determine whether the relationship between body size and metabolic rate differed among temperature treatments, data were visualised by plotting log $\dot{V}O_{2}$ against log size for each temperature at respective larval stages. Analysis of slopes for each temperature was conducted using *emmeans* and conducting Tukey‐adjusted pairwise contrasts with log‐size as variance.

To determine the effects of food and temperature on larval area, developmental stages were analysed separately. Data for early bipinnaria and brachiolaria were analysed by a type I one‐way ANOVA, with one fixed factor (temperature). The data for feeding stages of mid and late bipinnaria were analysed using a type I ANOVA with two fixed factors (feeding treatment and temperature). Assumptions of HOV and normality were met for all stages. Post hoc analyses were conducted using TukeyHSD.

All plots were generated using the R package *ggplot2* (Wickham 2016). For data visualisation, area‐corrected $\dot{V}O_{2}$ was plotted.

## 3 Results

### 3.1 Prefeeding Stages: Gastrula and Early Bipinnaria

**FIGURE 2:**
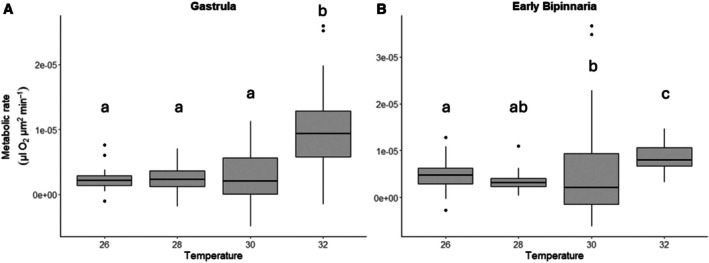
Respiration rates ($\dot{V}$O_2_) of *Acanthaster* cf. *solaris* gastrulae (~24 hpf) and early bipinnaria larvae (~72 hpf) determined as size specific metabolic rate (μLO_2_μm^2^ min^−1^) in control and elevated (28°C–32°C) temperatures. Boxplots show median $\dot{V}O_{2}$ (middle horizontal line), the interquartile $\dot{V}O_{2}$ range (rectangle) and range (vertical line/dots). Data points represent individual observations. (A) Gastrula respiration at temperatures 26°C (*n* = 64); 28°C (*n* = 32), 30°C (*n* = 32), 32°C (*n* = 32), (B) Early bipinnaria respiration at 26°C (*n* = 64); 28°C (*n* = 32), 30°C (*n* = 32), 32°C (*n* = 32). Treatments with the same letter were similar.

There was a significant effect of temperature on the metabolic rate of gastrulae (*χ* ^2^ = 46.43, *p* < 0.0001) with the 32°C treatment resulting in a higher metabolic rate than the other treatments which did not differ (Figure 2) (26°C = 28°C = 30°C < 32°C). In the 32°C treatment the mean metabolic rate was approximately 4‐fold higher than in the cooler treatments (Table 1).

**TABLE 1:** Mean respiration rates, surface area and standard error for *Acanthaster cf. solaris* across life stages, feeding regime and temperature treatments.

Stage	Feeding treatment	Temperature	Mean respiration (μLO_2_ min^−1^)	Standard error	Mean area (μm^2^)	Standard error
Gastrula	Prefeeding	26°C	2.54e‐05	0.37e‐05	—	—
		28°C	2.84e‐05	0.45e‐05	—	—
		30°C	2.94e‐05	0.69e‐05	—	—
		32°C	11.37e‐05	2.20e‐05	—	—
Early Bipinnaria	Prefeeding	26°C	6.36e‐05	0.66e‐05	1619	249
		28°C	5.55e‐05	0.59e‐05	1607	319
		30°C	8.76e‐05	2.73e‐05	1656	270
		32°C	11.17e‐05	0.65e‐05	1384	174
Mid Bipinnaria	Feeding	26°C	4.75e‐05	2.09e‐05	2466	129
		28°C	8.45e‐05	1.64e‐05	2066	96
		30°C	7.84e‐05	1.87e‐05	2287	534
		32°C	20.9e‐05	6.47e‐05	1934	500
Mid Bipinnaria	Unfed	26°C	2.09e‐05	4.54e‐05	1823	111
		28°C	6.01e‐05	6.58e‐05	2395	122
		30°C	13.1e‐05	11.1e‐05	2411	544
		32°C	19.0e‐5	11.1e‐05	1818	272
Late Bipinnaria	Feeding	26°C	11.6e‐05	2.47e‐05	3545	784
		28°C	6.06e‐05	1.08e‐05	2031	268
		30°C	5.30e‐05	1.33e‐05	1716	414
Late Bipinnaria	Unfed	26°C	8.14e‐05	8.46e‐05	1549	326
		28°C	4.28e‐05	1.31e‐05	1974	532
		30°C	4.45e‐05	4.09e‐05	2323	1335
Brachiolaria	Feeding	26°C	21.4e‐05	9.36e‐05	3477	748
		28°C	11.1e‐05	4.91e‐05	3674	1045

**FIGURE A3:**
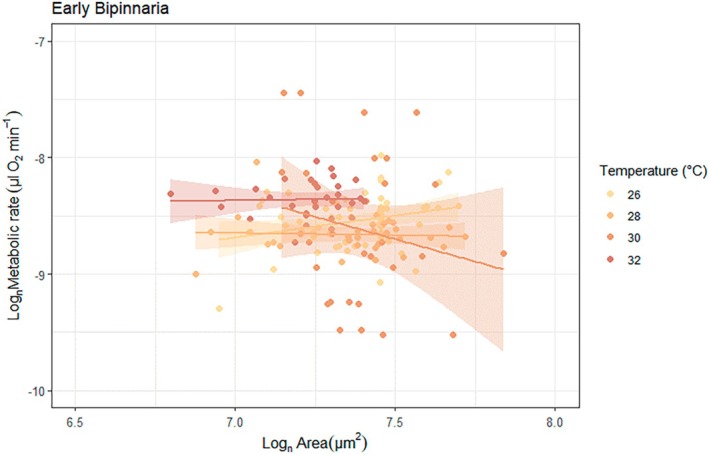
Natural log respiration rates (LogVO_2_) and size (Logμm^2^) of *Acanthaster* cf. *solaris* early bipinnaria larvae (~72 hpf) in control and elevated (28°C–32°C) temperatures.

There was no effect of temperature or size on the metabolic rate of the early bipinnaria larvae, but there was a significant interaction of temperature and size (Table 2, Figure A3). This was due to the influence of body size on metabolic rate at 30°C (*p* = 0.00591) and not in the other temperature treatments. Analysing larval area as a function of temperature indicated a significant effect on the size of early pre‐feeding bipinnaria larvae which were smaller in the 32°C treatments (Table 3). Metabolism of larvae reared at 30°C was significantly higher than those from the 26°C treatment, which was similar to that at 28°C, with the highest metabolism at 32°C (Figure 2). At 32°C, the mean metabolic rate was approximately 1.3–2 times higher than at lower temperatures (Table 1).

**TABLE 2:** Type III ANOVA analyses of log transformed metabolic rate data as a function of temperature and log transformed size for early bipinnaria (3 dpf) *Acanthaster cf. solaris*.

	Factor	df	DenDF	SS	F‐ratio	p‐value	Tukey HSD
Early Bipinnaria	Size	1	144	0.02587	0.4007	0.52772	26°C = 28°C = 30°C > 32°C
	Temperature	3	144	0.48256	2.4919	0.06256	30°C > 26°C = 28°C = 30°C < 32°C
	Size: Temperature	3	144	0.51909	2.6805	0.04917	—

**TABLE 3:** Type I ANOVA of size (μm^2^) data for *Acanthaster cf. solaris* bipinnaria and brachiolaria as a function of temperature and feeding regime.

	Factor	df	SS	F‐ratio	*p*	Tukey HSD
Early Bipinnaria	Temperature	3	1,533,790	7.5851	*p* < 0.001	26°C = 28°C = 30°C > 32°C
Mid Bipinnaria	Temperature	3	5,770,639	8.0340	*p* < 0.001	26°C = 32°C < 28°C = 30°C < 26°C = 28°C
	Food	1	254,263	1.0620	0.3044	Fed = Unfed
	Temperature * Food	3	36,392,566	9.5832	*p* < 0.001	26°C: fed>unfed; 28°C: fed = unfed; 30°C: fed = unfed; 32°C: fed = unfed
Late Bipinnaria	Temperature	2	11,596,294	26.846	*p* < 0.001	26°C > 28°C > 30°C
	Food	1	7,911,752	36.632	*p* < 0.001	Fed>Unfed
	Temperature * Food	2	24,325,401	56.314	*p* < 0.001	26°C: fed>unfed; 28°C: fed = unfed; 30°C: fed = unfed
Brachiolaria	Temperature	2	20,356,346	11.341	*p* < 0.001	30°C > 26°C < 28°C = 30°C

*Note:* Note for brachiolaria only fed larvae were available for measurement.

### 3.2 Feeding Stages: Mid Bipinnaria, Late Bipinnaria and Brachiolaria

**FIGURE 3:**
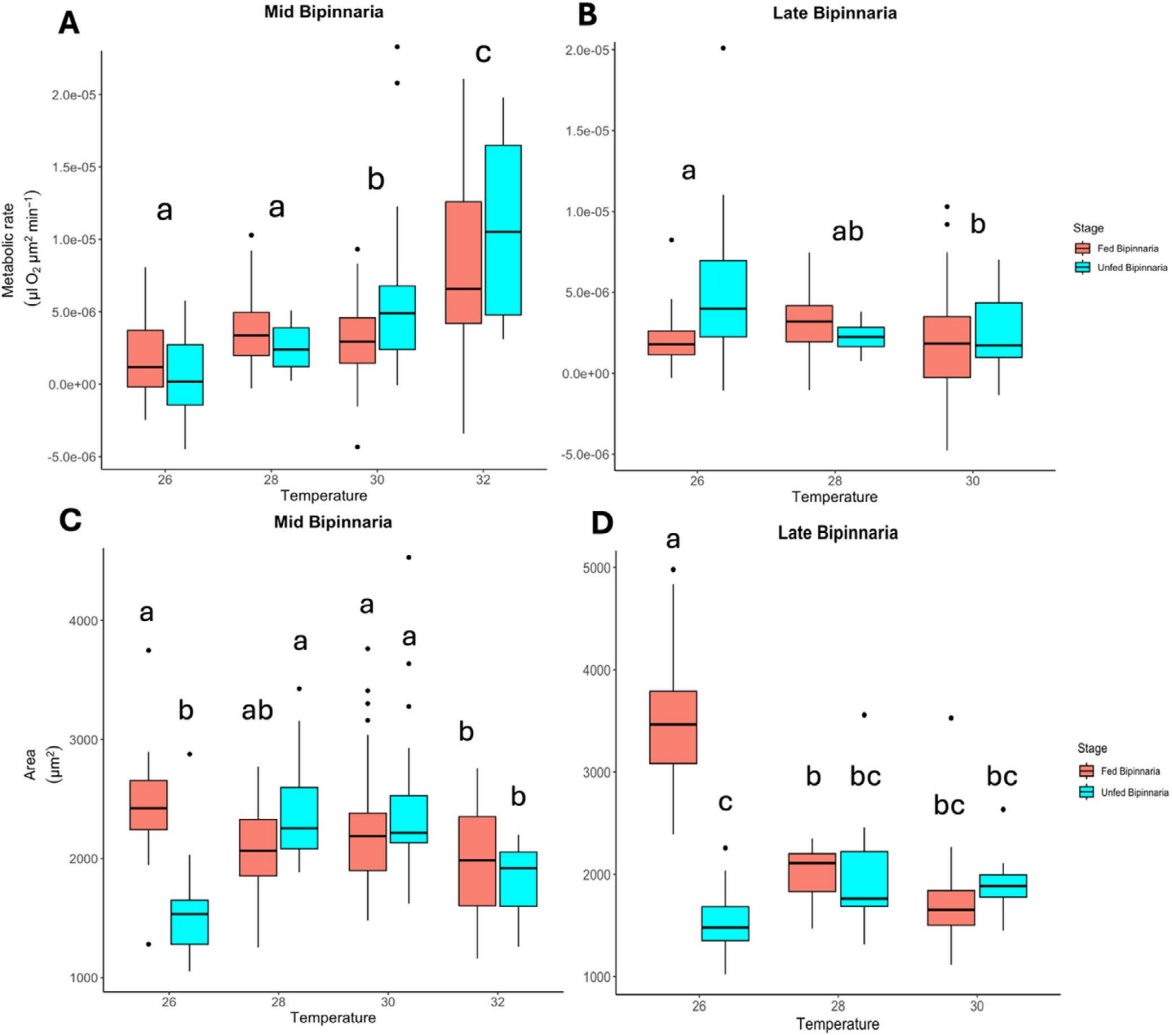
Respiration rates ($\dot{V}O_{2}$) determined as size specific metabolic rate (μLO_2_μm^2^min^−1^) and area (μm^2^) of *Acanthaster* cf. *solaris* fed and unfed mid bipinnaria (~7 dpf) and late bipinnaria (~14 dpf) in control and elevated (28°C–32°C and 28°C–30°C) temperatures. Boxplots show median $\dot{V}O_{2}$ (middle horizontal line), the interquartile $\dot{V}O_{2}$ range (rectangle) and range (vertical line/dots). Data points represent individual observations. The sample size ranged from 32 to 64. (A) mid bipinnaria respiration for fed and unfed larvae at 26°C (*n* = 16, fed and unfed); 28°C (*n* = 16, fed and unfed), 30°C (*n* = 32, fed and unfed), and 32°C (*n* = 16, fed and unfed). (B) late bipinnaria respiration for fed and unfed larvae at 26°C (*n* = 16, fed and unfed); 28°C (*n* = 16, fed and unfed), and 30°C (fed *n* = 16, unfed *n* = 14). (C) mid bipinnaria area for fed and unfed larvae at 26°C (fed *n* = 16, unfed *n* = 16); 28°C (fed *n* = 16, unfed *n* = 16), 30°C (fed *n* = 32, unfed *n* = 32), and 32°C (fed *n* = 16, unfed *n* = 16). (D) late bipinnaria area for fed and unfed larvae at 26°C (fed *n* = 16, unfed *n* = 16); 28°C (fed *n* = 16, unfed *n* = 16) and 30°C (fed *n* = 16, unfed *n* = 14). Treatments with the same letter were similar (fed and unfed treatments grouped by temperature for metabolic rates).

There was a significant effect of temperature on the metabolic rate of mid bipinnaria larvae, while size showed no effect (Table 4). The metabolic rates of larvae reared at 26°C were approximately 2.5–4.5 times lower than those in the 28°C, 30°C and 32°C treatments (Figure 3) (Table 1). Temperature affected the size of mid bipinnaria larvae but not feeding regime. Post hoc analysis found that there were significant differences in larval size with respect to food treatments at control (26°C), with no difference in the size of unfed and fed larvae in temperature treatments (Table 3).

**TABLE 4:** Type II ANOVA of metabolic rate data for *Acanthaster cf. solaris* as a function of temperature and larval size.

	Factor	df	DenDF	SS	F‐ratio	p‐value	Tukey HSD
Mid Bipinnaria	Size	1	155	0.1092	0.5659	0.453	26°C = 32°C < 28°C = 30°C < 26°C = 28°C
	Temperature	3	154.14	9.845	17.011	*p* < 0.001	26°C < 28°C = 30°C < 32°C
Late Bipinnaria	Size	1	103.27	0.0023	0.0214	0.883949	26°C > 28°C > 30°C
	Temperature	2	107.97	1.8640	8.6254	*p* < 0.001	28°C = 26°C > 30°C = 28°C
Brachiolaria	Size	1	49	0.4108	6.5963	0.01332	26°C > 28°C
	Temperature	1	49	0.2672	4.2902	0.04362	26°C > 28°C
	Size: Temperature	1	49	0.3078	4.9430	0.03084	—

*Note:* Interactions were run using AIC models (Tables A4 and A5).

For late bipinnaria, metabolism was only measured at 26°C, 28°C and 30°C. There was a significant effect of temperature but not food regime or size on metabolic rate (Table 4). Metabolic rate declined with increasing temperatures, with rates at 30°C approximately 50% lower than at 26°C. Post hoc analysis showed a significant difference in temperature treatments between 26°C and 30°C (Table 4) (Figure 3). Temperature and food treatment had a significant effect on size in late bipinnaria. There was an approximate 2‐fold increase in the size of fed larvae at 26°C (Table 1). For the fed treatment post hoc analysis found that late bipinnaria reared at 26°C were larger than those in warmer treatments (Post hoc: 26°C > 28°C > 30°C). The size of larvae in the unfed treatment did not differ across temperature treatments (Table 3).

**FIGURE 4:**
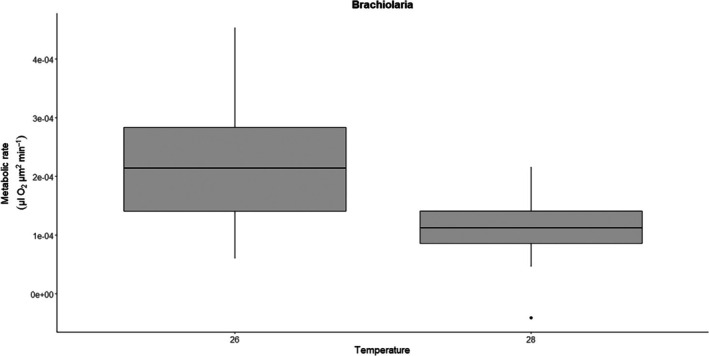
Respiration rates ($\dot{V}O_{2}$) of *Acanthaster* cf. *solaris* fed brachiolaria larvae determined as size specific metabolic rate (μLO_2_μm^2^min^−1^) in control and elevated (28°C) temperatures. Boxplots show median $\dot{V}O_{2}$ (middle horizontal line), the interquartile $\dot{V}O_{2}$ range (rectangle) and range (vertical line/dots). Data points represent individual observations. Data points represent individual observations. 26°C, *n* = 20, 28°C *n* = 32.

**FIGURE A4:**
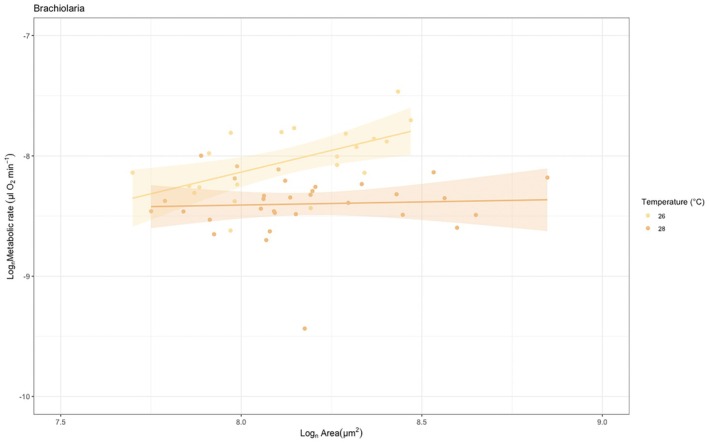
Natural log respiration rates (LogVO_2_) and size (Logμm^2^) of *Acanthaster* cf. *solaris* brachiolaria (~21 dpf) in control and elevated (28°C) temperatures.

For brachiolaria, temperature and size had a significant effect on metabolic rate (Figure 4) with a significant interaction between these factors seen in the positive relationship between body size and metabolic rate just in controls (26°C) (Figure A4). Post hoc comparison of slopes confirmed that there was a significant difference in larval size between temperatures (26°C > 28°C). Note, by the brachiolaria stage, only fed larvae were available for testing.

## 4 Discussion

Larval success is considered a key driver of population outbreaks of *Acanthaster* cf. *solaris* (Uthicke et al. 2015; Wolfe et al. 2015b; Deaker and Byrne 2022a) and here we show the metabolic resilience of the larvae even in no food conditions, a trait that may contribute to this success. The developmental stages had a higher metabolic rate at higher temperatures in their thermal tolerance range, similar to other marine ectotherms (Peck and Prothero‐Thomas 2002; Peck et al. 2008; Carey et al. 2016; Lang et al. 2023; Khalil et al. 2023). Importantly, CoTS larvae were able to maintain their metabolic rate for weeks while provisioned only by egg nutrients, pointing to metabolic resilience at the cost of nutritive condition. While higher metabolic rates at increased temperatures is likely to be beneficial to CoTS larvae due to less time spent in vulnerable larval stages, it is still contentious whether increased metabolic activity is correlated with increased fitness (Glazier 2015; Uthicke et al. 2015; Pettersen et al. 2016).

The presence of food promoted larval growth but only in the control temperature. Feeding also did not elicit a change in metabolic rate in any temperature treatment. For the warm treatments this suggests that exogenous food together with egg nutrients may compensate for temperature‐driven increased energy demands at the cost of enhancing nutritive condition and size. Fed larvae in the control 26°C treatment were able to increase their size indicating that given extra resources CoTS larvae can increase their nutritive condition, as seen in feeding larvae of asteroids and echinoids (Prowse et al. 2017). Interestingly, this was not the case for larvae reared in the higher temperature treatments. While maintaining a higher size‐specific metabolic rate to unfed larvae, those exposed to warmer temperatures did not increase their size. For these larvae it is not clear how the exogenous food was incorporated into metabolism.

Bipinnaria (14–21 days) and brachiolaria larvae in warm treatments (28°C and 30°C) had lower oxygen consumption, potentially indicating the onset of metabolic suppression as a mechanism to cope with thermal stress as suggested for other ectotherms (Guppy and Withers 1999). In addition, the presence of smaller larvae in the warm treatments may indicate selection for smaller size to facilitate metabolic requirements as well as suppression of metabolism in larger larvae (Atkinson 1995; Huey and Kingsolver 2019; Richard et al. 2024). Within ectotherm species, smaller individuals can display a higher thermal tolerance comparative to larger individuals (Pörtner 2002; Peck et al. 2009). That said, smaller larvae in the high temperature treatments may also simply reflect stunted growth due to the physiological impairment of cumulative heat stress over time.

Development of the initial functional feeding echinoderm larva typically exhausts egg nutrient reserves by ~3 dpf after which developmental arrest and mortality occur (Moran and Allen 2007; Prowse et al. 2017). In contrast, egg nutrients supported development of CoTS larvae well beyond this stage for up to two weeks. Possession of the large egg is a developmental resilience trait of CoTS (Caballes et al. 2016; Deaker et al. 2019). The FSW used for rearing would have had minimal nutrients likely reflecting the oligotrophic conditions that the larvae experience in their natural habitat. This indicates that the larvae are robust to these conditions. Beyond the late bipinnaria stage maternal nutrients did not support development to the settlement brachiolaria stage. Our results indicate that the FFP of CoTS larvae for when they can develop in the absence of exogenous food is ~19 days, after which development stalled and mortality ensued. This is similar to that reported previously (Pratchett et al. 2017; Mos et al. 2024). Beyond the late bipinnaria stage, in the absence of food, the larvae may have a negative metabolic budget and suffer ‘metabolic meltdown’ (Huey and Kingsolver 2019).

The presence of food did not result in an increase in metabolic rate in any treatment. While an increase might be expected (e.g., Rendleman et al. 2018; Ginsburg and Manahan 2009), the larvae were tested after the food had been cleared from their digestive track which may have reset metabolism to pre‐feeding levels as observed in gastropods and bryozoans (Crisp et al. 1978; Schuster et al. 2019). The lack of metabolic response post‐feeding after the food has been cleared suggests that the CoTS larvae do not maintain higher metabolic activity in response to feeding. This is in contrast to other echinoderm larvae which maintain a higher metabolic activity post feeding (Peck 1998; Peck et al. 2008; Ginsburg and Manahan 2009; Rendleman et al. 2018). Although CoTS larvae are known to be opportunistic feeders (Wolfe et al. 2015b; Uthicke et al. 2015), our results suggest that the presence of food does not increase metabolic rate.

As shown for fish larvae, individuals with higher levels of residual energy are generally better equipped to deal with environmental stress (Bochdansky et al. 2005). High levels of residual energy favour larvae with low standard metabolic rates, especially in environments where food supply is unpredictable, such as oligotrophic waters (Bochdansky et al. 2005). This is suggested to reflect natural selection towards low metabolic activity in these environments, build resilience to starvation and to capitalise on better conditions (Bochdansky et al. 2005). Being provisioned by high levels of maternal nutrients is likely to provide resilience to CoTS larvae in the face of environmental stress and thereby contribute to CoTS continued success (Uthicke et al. 2015; Caballes et al. 2016).

In nature CoTS larvae likely have access to a range of food including DOM, bacteria and low levels of phytoplankton typical of tropical waters (Nakajima et al. 2016; Mos et al. 2024). The presence of these sources of nutrition is likely to provide a buffer to help the larvae avoid ‘metabolic meltdown’. It will be important to understand how development proceeds in natural conditions to assess the relative contribution of maternal nutrients. A comprehensive understanding of the biochemistry of CoTS eggs and how egg nutrients are used by larvae is needed to understand the energy profile through development. This is also needed to identify the duration of the FFP of the larvae.

Despite the lack of difference in the metabolic rate of fed and unfed CoTS larvae—the fed larvae in control treatments were larger across all developmental stages. Larger fed larvae within thermal tolerance limits were expected as found in other studies (Lamare et al. 2014; Mos et al. 2024; Wolfe et al. 2015a). Previous studies also found that fed bipinnaria of CoTS reared at 30°C were smaller than those reared at 26°C treatments (Mos et al. 2023). Larvae reared at 30°C may struggle to maintain metabolic requirements, leading to a potential growth stasis as food is not offsetting metabolic costs and a selection towards smaller larvae. This potential growth stasis could be associated with changes to feeding behaviour in warm conditions, as seen in larvae of the sea star *Odontaster validus* , where prolonged thermal exposure caused larvae to stop feeding (Peck et al. 2008).

As ocean warming continues and marine heatwaves are becoming more frequent (*IPCC* 2023), CoTS larvae may benefit from increased metabolism in the warmer parts of its range. This metabolic increase would allow for a greater capacity for growth and development (Glazier 2015; Pettersen et al. 2016), with increased temperature reducing the time to larval settlement and thereby reducing the time spent in the plankton where predation is high (Vaughn and Allen 2010). As shown here for the larvae and previously for juveniles (Byrne et al. 2023), it appears that the early life stages of CoTS are more robust to warming than the coral prey of adult CoTS, which undergo bleaching and mortality in response to lower levels of warming than used here (Hughes et al. 2017; Byrne et al. 2025). In a warming world, the resilience of CoTS represents a threat for coral reefs already in a precarious state.

## 5 Conclusion

As the ocean warms, larval CoTS will have increased energetic costs in parallel with increased metabolism. This may promote larval success and thereby outbreak events. This success would be facilitated by their maternally provided nutritive buffer and shorter time in the plankton due to faster development. CoTS larvae appear to select for smaller individuals to be able to complete development in higher temperatures up to 30°C, but smaller larvae typically produce smaller juveniles which may compromise the success of the early benthic stage. Future research into the impacts of increased metabolic activity on CoTS development to the juvenile stage is needed to assess the potential impact of climate warming on CoTS fitness and population dynamics. How long the maternal nutrient buffer lasts in a potentially long FFP is important to quantify through energetic analysis. Given the trajectory of climate change and the threat that CoTS pose to coral reefs, understanding the effects of this increased metabolism on larvae will give insight into the population dynamics of CoTS in a warming ocean.

## Author Contributions

**Ronan T. Hill:** data curation (lead), formal analysis (lead), investigation (lead), methodology (equal), project administration (lead), visualization (equal), writing – original draft (lead), writing – review and editing (equal). **Amanda Pettersen:** conceptualization (equal), data curation (supporting), formal analysis (supporting), funding acquisition (equal), methodology (equal), resources (lead), software (lead), writing – review and editing (supporting). **Maria Byrne:** conceptualization (equal), funding acquisition (equal), methodology (equal), resources (equal), supervision (lead), validation (lead), writing – review and editing (lead).

## Funding

The authors have nothing to report.

## Disclosure

This research was conducted in Australia without discrimination on the basis of gender, ethnicity, nationality, cultural background or career stage. We acknowledge the importance of Aboriginal and Torres Strait Islander peoples' ongoing connection to Sea Country and recognise the value of Indigenous knowledge systems in understanding and managing marine ecosystems.

## Conflicts of Interest

The authors declare no conflicts of interest.

## Data Availability

The data in this manuscript are publicly available in the University of Sydney repository via the DOI https://doi.org/10.25910/1ny6‐s981.

## Appendix A

**TABLE A1:** Number of *Acanthaster cf. solaris* larvae used in respiration measurements at each respective life stage.

Stage	Feeding treatment	Temperature	Number measured
Gastrula	Prefeeding	26°C	32
		28°C	32
		30°C	32
		32°C	32
Early Bipinnaria	Prefeeding	26°C	64
		28°C	32
		30°C	32
		32°C	32
Mid Bipinnaria	Feeding	26°C	16
		28°C	16
		30°C	32
		32°C	16
Mid Bipinnaria	Unfed	26°C	16
		28°C	16
		30°C	32
		32°C	16
Late Bipinnaria	Feeding	26°C	16
		28°C	16
		30°C	32
Late Bipinnaria	Unfed	26°C	16
		28°C	16
		30°C	16
Brachiolaria	Feeding	26°C	21
		28°C	32

**TABLE A2:** Type III ANOVA with Satterthwaite's method analyses of metabolic rate data for *Acanthaster cf. solaris* feeding stages as a function of temperature, feeding regime and larval size.

	Factor	df	DenDF	SS	F‐ratio	*p*	Tukey HSD
Mid Bipinnaria	Size	1	153.45	0.0993	0.5311	0.46756	26°C: fed>unfed; 28°C: fed<unfed; 30°C: fed = unfed; 32°C: fed = unfed
	Temperature	3	153.05	9.9449	17.7376	*p* < 0.001	26°C < 28°C = 30°C < 32°C
	Food	1	132.43	0.9795	5.2411	0.02367	26°C: fed = unfed; 28°C: fed = unfed; 30°C: fed = unfed; 32°C: fed = unfed
Late Bipinnaria	Size	1	106.40	0.0117	0.1084	0.7426603	26°C: fed>unfed; 28°C: fed = unfed; 30°C: fed = unfed
	Temperature	2	106.67	1.6509	7.6445	*p* < 0.001	28°C = 26°C > 30°C = 28°C
	Food	1	107	0.0956	0.8850	0.3489640	26°C: fed = unfed; 28°C: fed = unfed; 30°C: fed = unfed; 32°C: fed = unfed

**TABLE A3:** Summary of candidate models used in model selection log_n_ metabolic rate for non‐feeding stages of crown‐of‐thorns larvae. Fixed predictor variables were log_n_ area and temperature (26°C, 28°C, 30°C and 32°C). Sensor dish plate was included as a random effect for all models. Parameters that have a significant slope are in bold.

Predictors	AIC	Diff AIC	AIC_wt_	Sum of AIC_wt_	*R* ^2^
**Temperature**	220.6	0.00	0.598	0.598	0.094
LogArea	225.5	4.94	0.050	0.648	0.018
LogArea + Temperature	223.8	3.19	0.121	0.769	0.095
LogArea *** Temperature**	222.5	1.90	0.231	1.0	0.146

**TABLE A4:** Summary of candidate models used in model selection log_n_ metabolic rate for middle bipinnaria of crown‐of‐thorns larvae. Fixed predictor variables were log_n_ area, temperature (26°C, 28°C, 30°C and 32°C) and treatment (fed or unfed). Sensor dish plate was included as a random effect for all models. Parameters that have a significant slope are in bold.

Predictors	AIC	Diff AIC	AIC_wt_	Sum of AIC_wt_	*R* ^2^
**Temperature**	214	0.00	0.467	0.467	0.266
logArea	247.3	33.26	0	0.467	0.021
**Treatment**	245.7	31.71	0	0.467	0.120
**Temperature** + LogArea	217.6	3.62	0.077	0.544	0.264
**Temperature *** LogArea	221.5	7.54	0.011	0.555	0.270
**Temperature** + **Treatment**	214.6	0.64	0.339	0.894	0.356
**Temperature * Treatment**	221.1	7.15	0.013	0.907	0.328
LogArea + **Treatment**	249.4	35.43	0	0.907	0.116
LogArea *** Treatment**	251.6	37.61	0	0.907	0.106
LogArea + **Temperature** + **Treatment**	218.3	4.34	0.053	0.960	0.351
LogArea *** Temperature** + **Treatment**	222.8	8.79	0.006	0.966	0.343
LogArea * **Treatment** + **Temperature**	219.3	5.32	0.033	0.999	0.378
**Temperature** * **Treatment** + LogArea	225.3	11.26	0.001	1.0	0.326
LogArea *** Temperature * Treatment**	229.7	15.73	0	1.0	0.356

**TABLE A5:** Summary of candidate models used in model selection log_n_ metabolic rate for late bipinnaria of crown‐of‐thorns larvae. Fixed predictor variables were log_n_ area, temperature (26°C, 28°C and 30°C) and treatment (fed or unfed). Sensor dish plate was included as a random effect for all models. Parameters that have a significant slope are in bold.

Predictors	AIC	Diff AIC	AIC_wt_	Sum of AIC_wt_	*R* ^2^
**Temperature**	89.4	0.00	0.819	0.819	0.266
LogArea	98.5	9.17	0.008	0.828	0.209
**Treatment**	102.7	13.37	0.001	0.829	0.120
**Temperature** + **LogArea**	94	4.68	0.079	0.908	0.264
**Temperature** * LogArea	98.6	9.25	0.008	0.916	0.270
**Temperature** + **Treatment**	94.3	4.97	0.068	0.984	0.355
**Temperature * Treatment**	101.3	11.98	0.002	0.986	0.328
LogArea + **Treatment**	102	12.66	0.001	0.987	0.116
LogArea *** Treatment**	100.3	10.92	0.003	0.990	0.106
LogArea + **Temperature** + **Treatment**	98.9	9.55	0.007	0.997	0.351
LogArea *** Temperature** + **Treatment**	102.2	12.87	0.001	0.998	0.343
**LogArea * Treatment** + **Temperature**	101.7	12.37	0.002	1	0.378
**Temperature * Treatment** + LogArea	105.4	16.06	0	1	0.326
LogArea *** Temperature * Treatment**	111.3	21.96	0	1	0.356

## References

[ece373934-bib-0001] Atkinson, D. 1995. “Effects of Temperature on the Size of Aquatic Ectotherms: Exceptions to the General Rule.” Journal of Thermal Biology 20, no. 1: 61–74. 10.1016/0306-4565(94)00028-H.

[ece373934-bib-0002] Bartoń, K. 2025. MuMIn: Multi‐Model Inference. https://CRAN.R‐project.org/package=MuMIn.

[ece373934-bib-0003] Bates, D. , M. Mächler , B. Bolker , and S. Walker . 2015. “Fitting Linear Mixed‐Effects Models Using lme4.” Journal of Statistical Software 67, no. 1: 1–48. 10.18637/jss.v067.i01.

[ece373934-bib-0004] Bochdansky, A. B. , P. Grønkjær , T. P. Herra , and W. C. Leggett . 2005. “Experimental Evidence for Selection Against Fish Larvae With High Metabolic Rates in a Food Limited Environment.” Marine Biology 147, no. 6: 1413–1417. 10.1007/s00227-005-0036-z.

[ece373934-bib-0005] Brockington, S. , and A. Clarke . 2001. “The Relative Influence of Temperature and Food on the Metabolism of a Marine Invertebrate.” Journal of Experimental Marine Biology and Ecology 258, no. 1: 87–99. 10.1016/S0022-0981(00)00347-6. 11239627

[ece373934-bib-0006] Byrne, M. , D. J. Deaker , M. Gibbs , P. Selvakumaraswarmy , and M. Clements . 2023. “Juvenile Waiting Stage Crown‐Of‐Thorns Sea Stars Are Resilient in Heatwave Conditions That Bleach and Kill Corals.” Global Change Biology 29, no. 23: 6493–6502. 10.1111/gcb.16946. 37849435

[ece373934-bib-0007] Byrne, M. , M. L. Gall , H. Campbell , M. D. Lamare , and S. P. Holmes . 2022. “Staying in Place and Moving in Space: Contrasting Larval Thermal Sensitivity Explains Distributional Changes of Sympatric Sea Urchin Species to Habitat Warming.” Global Change Biology 28, no. 9: 3040–3053. 10.1111/gcb.16116. 35108424

[ece373934-bib-0008] Byrne, M. , M. A. Sewell , and T. A. A. Prowse . 2008. “Nutritional Ecology of Sea Urchin Larvae: Influence of Endogenous and Exogenous Nutrition on Echinopluteal Growth and Phenotypic Plasticity in Tripneustes Gratilla.” Functional Ecology 22: 643–648. 10.1111/j.1365-2435.2008.01427x.

[ece373934-bib-0079] Byrne, M. , A. Waller , C. Matthew , et al. 2025. “Catastrohpic Bleaching in Protected Reefs of the Southern Great Barrier Reef.” Limnology and Oceanography 10, no. 3: 340–348. 10.1002/lol2.10456.

[ece373934-bib-0009] Caballes, C. F. , M. S. Pratchett , A. M. Kerr , and J. A. Rivera‐Posada . 2016. “The Role of Maternal Nutrition on Oocyte Size and Quality, With Respect to Early Larval Development in the Coral‐Eating Starfish, *Acanthaster planci* .” PLoS One 11, no. 6: e0158007. 10.1371/journal.pone.0158007. 27327627 PMC4915722

[ece373934-bib-0010] Cameron, J. N. 1986. “The Solubility of Carbon Dioxide as a Function of Temperature and Salinity (Appendix Table).” In Principles of Physiological Measurement, edited by J. N. Cameron , 254–259. Academic Press.

[ece373934-bib-0011] Carey, N. , J. Harianto , and M. Byrne . 2016. “Sea Urchins in a High‐CO2 World: Partitioned Effects of Body Size, Ocean Warming and Acidification on Metabolic Rate.” Journal of Experimental Biology 219: 1178–1186. 10.1242/jeb.136101. 26896541

[ece373934-bib-0012] Clements, M. , P. Selvakumaraswamy , D. Deaker , and M. Byrne . 2022. “Freshening of Great Barrier Reef Waters Is Deleterious for Larval Crown‐Of‐Thorns Starfish, Counter to the Terrestrial Runoff Hypothesis.” Marine Ecology Progress Series 696: 1–14. 10.3354/meps14150.

[ece373934-bib-0013] Crisp, M. , J. Davenport , and S. E. Shumway . 1978. “Effects of Feeding and of Chemical Stimulation on the Oxygen Uptake of *Nassarius reticulatus* (Gastropoda: Prosobranchia).” Journal of the Marine Biological Association of the United Kingdom 58, no. 2: 387–399. 10.1017/S002531540002806X.

[ece373934-bib-0014] Deaker, D. J. , and M. Byrne . 2022a. “Crown of Thorns Starfish Life‐History Traits Contribute to Outbreaks, a Continuing Concern for Coral Reefs.” Emerging Topics in Life Sciences 6, no. 1: 67–79. 10.1042/ETLS20210239. 35225331 PMC9023020

[ece373934-bib-0015] Deaker, D. J. , and M. Byrne . 2022b. “The Relationship Between Size and Metabolic Rate of Juvenile Crown of Thorns Starfish.” Invertebrate Biology 141, no. 3: e12382. 10.1111/ivb.12382.

[ece373934-bib-0016] Deaker, D. J. , S. A. Foo , and M. Byrne . 2019. “Variability in Egg and Jelly‐Coat Size and Their Contribution to Target Size for Spermatozoa: A Review for the Echinodermata.” Marine and Freshwater Research 70, no. 7: 995–1006. 10.1071/MF18134.

[ece373934-bib-0017] Foo, S. A. , H. R. Millican , and M. Byrne . 2024. “Crown‐Of‐Thorns Seastar (Acanthaster spp.) Feeding Ecology Across Species and Regions.” Sci Total Environ 930: 172691. 10.1016/j.scitotenv.2024.172691. 38663591

[ece373934-bib-0018] Ginsburg, D. W. , and D. T. Manahan . 2009. “Developmental Physiology of Antarctic Asteroids With Different Life‐History Modes.” Marine Biology 156, no. 11: 2391–2402. 10.1007/s00227-009-1268-0.

[ece373934-bib-0019] Glazier, D. S. 2015. “Is Metabolic Rate a Universal “Pacemaker” for Biological Processes?” Biological Reviews 90, no. 2: 377–407. 10.1111/brv.12115. 24863680

[ece373934-bib-0020] Guppy, M. , and P. Withers . 1999. “Metabolic Depression in Animals: Physiological Perspectives and Biochemical Generalizations.” Biological Reviews 74, no. 1: 1–40. 10.1017/S0006323198005258. 10396183

[ece373934-bib-0021] Hardison, E. A. , and E. J. Eliason . 2024. “Diet Effects on Ectotherm Thermal Performance.” Biological Reviews 99, no. 4: 1537–1555. 10.1111/brv.13081. 38616524

[ece373934-bib-0022] Harianto, J. , N. Carey , M. Byrne , and S. Price . 2019. “respR—An R Package for the Manipulation and Analysis of Respirometry Data.” Methods in Ecology and Evolution 10, no. 6: 912–920. 10.1111/2041-210X.13162.

[ece373934-bib-0023] Harianto, J. , H. D. Nguyen , S. P. Holmes , and M. Byrne . 2018. “The Effect of Warming on Mortality, Metabolic Rate, Heat‐Shock Protein Response and Gonad Growth in Thermally Acclimated Sea Urchins (Heliocidaris Erythrogramma).” Marine Biology 165, no. 6: 1–12. 10.1007/s00227-018-3353-8.

[ece373934-bib-0024] Hartig, F. 2024. DHARMa: Residual Diagnostics for Hierarchical (Multi‐Level/Mixed) Regression Models. https://CRAN.R‐project.org/package=DHARMa.

[ece373934-bib-0025] Haszprunar, G. , and M. Spies . 2014. “An Integrative Approach to the Taxonomy of the Crown‐Of‐Thorns Starfish Species Group (Asteroidea: Acanthaster): A Review of Names and Comparison to Recent Molecular Data.” Zootaxa 3481: 271–284. 10.11646/zootaxa.3481.2.6. 25082040

[ece373934-bib-0026] Haure, J. , A. Huvet , H. Palvadeau , et al. 2003. “Feeding and Respiratory Time Activities in the Cupped Oysters *Crassostrea gigas* , Crassostrea Angulata and Their Hybrids.” Aquaculture 218, no. 1: 539–551. 10.1016/S0044-8486(02)00493-3.

[ece373934-bib-0027] Huey, R. B. , and J. B. Kingsolver . 2019. “Climate Warming, Resource Availability, and the Metabolic Meltdown of Ectotherms.” American Naturalist 194, no. 6: E140–E150. 10.1086/705679. 31738103

[ece373934-bib-0028] Hughes, T. P. , J. T. Kerry , M. Álvarez‐Noriega , et al. 2017. “Global Warming and Recurrent Mass Bleaching of Corals.” Nature 543, no. 7645: 373–377. 10.1038/nature21707. 28300113

[ece373934-bib-0029] Hutchings, P. , C. E. Rowe , M. Byrne , and R. Przeslawski . 2025. “Taxonomy Is a Foundation of Marine Science, and It Is in Trouble.” Advances in Marine Biology 101: 198–212. 10.1016/bs.amb.2025.08.003 41162143

[ece373934-bib-0030] IPCC . 2023. Climate Change 2023: Synthesis Report. Contribution of Working Groups I, II and III to the Sixth Assessment Report of the Intergovernmental Panel on Climate Change Core Writing Team, edited by H. Lee and J. Romero , 184. IPCC. 10.59327/IPCC/AR6-9789291691647.

[ece373934-bib-0031] Irwin, S. , and J. Davenport . 2006. “Implications of Water Flow and Oxygen Gradients for Molluscan Oxygen Uptake and Respirometric Measurements.” Journal of the Marine Biological Association of the UK 86: 401–402. 10.1017/S0025315406013257.

[ece373934-bib-0032] Kamya, P. Z. , M. Byrne , A. Graba‐Landry , and S. A. Dworjanyn . 2016. “Near‐Future Ocean Acidification Enhances the Feeding Rate and Development of the Herbivorous Juveniles of the Crown‐Of‐Thorns Starfish, ‘ *Acanthaster planci* ’.” Coral Reefs 35, no. 4: 1241–1251. 10.1007/s00338-016-1480-6.

[ece373934-bib-0033] Kamya, P. Z. , S. A. Dworjanyn , N. Hardy , B. Mos , S. Uthicke , and M. Byrne . 2014. “Larvae of the Coral Eating Crown‐Of‐Thorns Starfish, *Acanthaster planci* in a Warmer‐High CO2 Ocean.” Global Change Biology 20: 3365–3376. 10.1111/gcb.12530. 24615941

[ece373934-bib-0034] Khalil, M. , S. S. Doo , M. Stuhr , and H. Westphal . 2023. “Long‐Term Physiological Responses to Combined Ocean Acidification and Warming Show Energetic Trade‐Offs in an Asterinid Starfish.” Coral Reefs 42, no. 4: 845–858. 10.1007/s00338-023-02388-2.

[ece373934-bib-0035] Köster, M. , C. Krause , and G.‐A. Paffenhöfer . 2008. “Time Series Measurements of Oxygen Consumption of Copepod Nauplii.” Marine Ecology Progress Series 353: 157–164. 10.3354/meps07185.

[ece373934-bib-0036] Lamare, M. , D. Pecorino , N. Hardy , M. Liddy , M. Byrne , and S. Uthicke . 2014. “The Thermal Tolerance of Crown‐Of‐Thorns ( *Acanthaster planci* ) Embryos and Bipinnaria Larvae: Implications for Spatial and Temporal Variation in Adult Populations.” Coral Reefs 33: 207–219. 10.1007/s00338-013-1112-3.

[ece373934-bib-0037] Lang, B. J. , J. M. Donelson , K. R. Bairos‐Novak , et al. 2023. “Impacts of Ocean Warming on Echinoderms: A Meta‐Analysis.” Ecology and Evolution 13, no. 8: e10307. 10.1002/ece3.10307. 37565029 PMC10409743

[ece373934-bib-0038] Lee, T. H. , R. A. R. McGill , and S. Fitzer . 2021. “Effects of Extra Feeding Combined With Ocean Acidification and Increased Temperature on the Carbon Isotope Values (δ13C) in the Mussel Shell.” Journal of Experimental Marine Biology and Ecology 541: 151562. 10.1016/j.jembe.2021.151562.

[ece373934-bib-0039] Lenth, R. 2025. Emmeans: Estimated Marginal Means, AKA Least‐Square Means.

[ece373934-bib-0040] McEdward, L. R. 1985. “Effects of Temperature on the Body Form, Growth, Electron Transport System Activity, and Developmental Rate of an Echinopluteus.” Journal of Experimental Marine Biology and Ecology 93: 163–181. 10.1016/0022-0981(85)90157-1.

[ece373934-bib-0041] McGeady, R. , C. Lordan , and A. M. Power . 2021. “Shift in the Larval Phenology of a Marine Ectotherm due to Ocean Warming With Consequences for Larval Transport.” Limnology and Oceanography 66, no. 2: 543–557. 10.1002/lno.11622.

[ece373934-bib-0042] Moran, A. L. , and J. D. Allen . 2007. “How Does Metabolic Rate Scale With Egg Size? An Experimental Test With Sea Urchin Embryos.” Biological Bulletin 212, no. 2: 143–150. 10.2307/25066591. 17438206

[ece373934-bib-0043] Moreira, J. M. , A. C. Mendes , A. L. Maulvault , et al. 2022. “Impacts of Ocean Warming and Acidification on the Energy Budget of Three Commercially Important Fish Species.” Conservation Physiology 10, no. 1: 1. 10.1093/conphys/coac048. PMC9305255 35875680

[ece373934-bib-0044] Mos, B. , D. Erler , C. Lawson , and S. A. Dworjanyn . 2024. “Crown‐Of‐Thorns Starfish Complete Their Larval Phase Eating Only Nitrogen‐Fixing Trichodesmium Cyanobacteria.” Science Advances 10: eado2682. 10.1126/sciadvado2682. 39018391 PMC466945

[ece373934-bib-0045] Mos, B. , N. Mesic , and S. A. Dworjanyn . 2023. “Variable Food Alters Responses of Larval Crown‐Of‐Thorns Starfish to Ocean Warming but Not Acidification.” Communications Biology 6, no. 1: 639. 10.1038/s42003-023-05028-1. 37316528 PMC10267210

[ece373934-bib-0046] Munstermann, M. J. , S. E. Karelitz , R. Ferraro , L. Rogers‐Bennett , R. D. Simons , and D. K. Okamoto . 2026. “Food Limitation Erodes the Thermal Tolerance of Larvae in an Ecologically Influential Marine Herbivore.” Ecology 107, no. 1: e70288. 10.1002/ecy.70288. 41555599 PMC12816818

[ece373934-bib-0047] Nakajima, R. , N. Nakatomi , H. Kurihara , M. D. Fox , J. E. Smith , and K. Okaji . 2016. “Crown‐Of‐Thorns Starfish Larvae Can Feed on Organic Matter Released From Corals.” Diversity 8, no. 4: 18. 10.3390/d8040018.

[ece373934-bib-0048] Olson, R. R. 1987. “In Situ Culturing as a Test of the Larval Starvation Hypothesis for the Crown‐Of‐Thorns Starfish, *Acanthaster planci* .” Limnology and Oceanography 32, no. 4: 895–904.

[ece373934-bib-0049] Pace, D. A. , and D. T. Manahan . 2007. “Efficiencies and Costs of Larval Growth in Different Food Environments (Asteroidea: *Asterina miniata* ).” Journal of Experimental Marine Biology and Ecology 353, no. 1: 89–106. 10.1016/j.jembe.2007.09.005.

[ece373934-bib-0050] Patel, F. , C. Zeng , M. Logan , and S. Uthicke . 2024. “Feeding Rates and Carbon and Nitrogen Partitioning in Crown‐Of‐Thorns Sea Star Larvae (Acanthaster Cf. Solaris) During Development.” Marine Biology 171, no. 2: 61. 10.1007/s00227-023-04377-z.

[ece373934-bib-0052] Peck, L. S. 1998. “Feeding, Metabolism and Metabolic Scope in Antarctic Marine Ectotherms.” In Cold Ocean Physiollgy, edited by H. O. Pörtner and R. C. Playle , vol. 66, 365–390. Soc Exp Biol Sem Ser.

[ece373934-bib-0053] Peck, L. S. , M. S. Clark , S. A. Morley , A. Massey , and H. Rossetti . 2009. “Animal Temperature Limits and Ecological Relevance: Effects of Size, Activity and Rates of Change.” Functional Ecology 23, no. 2: 248–256. 10.1111/j.1365-2435.2008.01537x.

[ece373934-bib-0054] Peck, L. S. , and E. Prothero‐Thomas . 2002. “Temperature Effects on the Metabolism of Larvae of the Antarctic Starfish *Odontaster validus* , Using a Novel Micro‐Respirometry Method.” Marine Biology 141, no. 2: 271–276. 10.1007/s00227-002-0834-5.

[ece373934-bib-0051] Peck, L. , K. Webb , A. Miller , M. Clark , and T. Hill . 2008. “Temperature Limits to Activity, Feeding and Metabolism in the Antarctic Starfish *Odontaster validus* .” Marine Ecology Progress Series 358: 181–189. 10.3354/meps07336.

[ece373934-bib-0055] Pettersen, A. K. , D. J. Marshall , and C. R. White . 2018. “Understanding Variation in Metabolic Rate.” Journal of Experimental Biology 221: jeb166876. 10.1242/jeb.166876. 29326115

[ece373934-bib-0056] Pettersen, A. K. , C. R. White , and D. J. Marshall . 2015. “Why Does Offspring Size Affect Performance? Integrating Metabolic Scaling With Life‐History Theory.” Proceedings of the Royal Society B 282, no. 1819: 20151946. 10.1098/rspb.2015.1946. 26559952 PMC4685814

[ece373934-bib-0057] Pettersen, A. K. , C. R. White , and D. J. Marshall . 2016. “Metabolic Rate Covaries With Fitness and the Pace of the Life History in the Field.” Proceedings of the Royal Society B 283: 20160323. 10.1098/rspb.2016.0323. 27226476 PMC4892794

[ece373934-bib-0058] Pörtner, H. O. 2002. “Physiological Basis of Temperature‐Dependent Biogeography: Trade‐Offs in Muscle Design and Performance in Polar Ectotherms.” Journal of Experimental Biology 205, no. 15: 2217–2230. 10.1242/jeb.205.15.2217. 12110656

[ece373934-bib-0059] Pörtner, H. O. , and R. Knust . 2007. “Climate Change Affects Marine Fishes Through the Oxygen Limitation of Thermal Tolerance.” Science 315: 95–97. 10.1002/evl3.174. 17204649

[ece373934-bib-0060] Pottier, P. , N. C. Wu , M. L. Earhart , et al. 2026. “Embryos Are Largely Understudied in a Representative Sample of Journals in Conservation Physiology.” Conservation Physiology 14, no. 1: coag006. 10.1093/conphys/coag006. 41727255 PMC12916238

[ece373934-bib-0062] Pratchett, M. S. , C. F. Caballes , J. Rivera Posada , and H. Sweatman . 2014. “Limits to Understanding and Managing Outbreaks of Crown‐Of‐Thorns Starfish (Acanthaster spp).” In Oceanography and Marine Biology, 133–200. CRC Press. 10.1201/b17143-4.

[ece373934-bib-0061] Pratchett, M. , S. Dworjanyn , B. Mos , C. Caballes , C. Thompson , and S. Blowes . 2017. “Larval Survivorship and Settlement of Crown‐Of‐Thorns Starfish (Acanthaster Cf. Solaris) at Varying Algal Cell Densities.” Diversity 9, no. 1: 2. 10.3390/d9010002.

[ece373934-bib-0063] Prowse, T. A. A. , M. A. Sewell , and M. Byrne . 2017. “Three‐Stage Lipid Dynamics During Development of Planktotrophic Echinoderm Larvae.” Marine Ecology Progress Series 583: 149–161. 10.3354/meps12335.

[ece373934-bib-0064] Przeslawski, R. , M. Byrne , and C. Mellin . 2015. “A Review and Meta‐Analysis of the Effects of Multiple Abiotic Stressors on Marine Embryos and Larvae.” Global Change Biology 21: 2122–2140. 25488061 10.1111/gcb.12833

[ece373934-bib-0065] Rendleman, A. J. , J. A. Rodriguez , A. Ohanian , and D. A. Pace . 2018. “More Than Morphology: Differences in Food Ration Drive Physiological Plasticity in Echinoid Larvae.” Journal of Experimental Marine Biology and Ecology 501: 1–15. 10.1016/j.jembe.2017.12.018.

[ece373934-bib-0066] Richard, R. , J. A. Lugsanay , and S. P. Huang . 2024. “The Temperature‐Size Rule in the Context of Dynamic Energy Budget Theory.” Ecological Modelling 493: 110761. 10.1016/j.ecolmodel.2024.110761.

[ece373934-bib-0067] Rohr, J. R. , D. J. Civitello , J. M. Cohen , et al. 2018. “The Complex Drivers of Thermal Acclimation and Breadth in Ectotherms.” Ecology Letters 21, no. 9: 1425–1439. 10.1111/ele.13107. 30009486

[ece373934-bib-0068] Schuster, L. , C. R. White , and D. J. Marshall . 2019. “Influence of Food, Body Size, and Fragmentation on Metabolic Rate in a Sessile Marine Invertebrate.” Invertebrate Biology 138, no. 1: 55–66. 10.1111/ivb.12241.

[ece373934-bib-0069] Schwemmer, T. G. , H. Baumann , C. S. Murray , A. I. Molina , and J. A. Nye . 2020. “Acidification and Hypoxia Interactively Affect Metabolism in Embryos, but Not Larvae, of the Coastal Forage Fish *Menidia menidia* .” Journal of Experimental Biology 223, no. PT 22. 10.1242/jeb.228015. 33046569

[ece373934-bib-0070] Somero, G. N. 2012. “The Physiology of Global Change: Linking Patterns to Mechanisms.” Annual Review of Marine Science 4, no. 1: 39–61. 10.1146/annurev-marine-120710-100935. 22457968

[ece373934-bib-0071] Sunday, J. M. , A. E. Bates , and N. K. Dulvy . 2011. “Global Analysis of Thermal Tolerance and Latitude in Ectotherms.” Proceedings of the Royal Society B 278, no. 1713: 1823–1830. 10.1098/rspb.2010.1295. 21106582 PMC3097822

[ece373934-bib-0072] Tyrell, A. S. , N. S. Fisher , and D. M. Fields . 2020. “Separating Thermal and Viscous Effects of Temperature on Copepod Respiration and Energy Budget.” Biological Bulletin 239, no. 1: 62–71. 10.1086/709646. 32812813

[ece373934-bib-0073] Uthicke, S. , M. Logan , M. Liddy , D. Francis , N. Hardy , and M. Lamare . 2015. “Climate Change as an Unexpected Co‐Factor Promoting Coral Eating Seastar ( *Acanthaster planci* ) Outbreaks.” Scientific Reports 5, no. 1: 8402. 10.1038/srep08402. 25672480 PMC4325318

[ece373934-bib-0074] Vaughn, D. , and J. D. Allen . 2010. “The Peril of the Plankton.” Integrative and Comparative Biology 50, no. 4: 552–570. 10.1093/icb/icq037. 21558224

[ece373934-bib-0075] Vorsatz, L. D. , P. Pattrick , and F. Porri . 2021. “Fine‐Scale Conditions Across Mangrove Microhabitats and Larval Ontogeny Contributes to the Thermal Physiology of Early Stage Brachyurans (Crustacea: Decapoda).” Conservation Physiology 9, no. 1: coab010. 10.1093/conphys/coab010. 33927883 PMC8059134

[ece373934-bib-0076] Wickham, H. 2016. ggplot2: Elegant Graphics for Data Analysis. Springer‐Verlag New York. https://ggplot2.tidyverse.org.

[ece373934-bib-0077] Wolfe, K. , A. Graba‐Landry , S. A. Dworjanyn , and M. Byrne . 2015a. “Larval Starvation to Satiation: Influence of Nutrient Regime on the Success of *Acanthaster planci* .” PLoS One 10, no. 3: e0122010. 10.1371/journal.pone.0122010. 25790074 PMC4366153

[ece373934-bib-0078] Wolfe, K. , A. Graba‐Landry , S. A. Dworjanyn , and M. Byrne . 2015b. “Larval Phenotypic Plasticity in the Boom‐And‐Bust Crown‐Of‐Thorns Seastar, *Acanthaster planci* .” Marine Ecology Progress Series 539: 179–189. 10.3354/meps11495.

